# Seasonality Influence on Biochemical and Hematological Indicators of Stress and Growth of Pirarucu (*Arapaima gigas*), an Amazonian Air-Breathing Fish

**DOI:** 10.1155/2014/541278

**Published:** 2014-01-21

**Authors:** Rosiely Felix Bezerra, Maria do Carmo Figueiredo Soares, Athiê Jorge Guerra Santos, Elba Verônica Matoso Maciel Carvalho, Luana Cassandra Breitenbach Barroso Coelho

**Affiliations:** ^1^Universidade Federal de Pernambuco (UFPE), Centro de Ciências Biológicas, Departamento de Bioquímica, Rua Professor Nelson Chaves s/n, Cidade Universitária, 50670-901 Recife, PE, Brazil; ^2^Universidade Federal Rural de Pernambuco (UFRPE), Departamento de Engenharia de Pesca, Rua Dom Manoel de Medeiros, s/n, Dois Irmãos, 52171-900 Recife, PE, Brazil

## Abstract

Environmental factors such as seasonal cycles are the main chronic stress cause in fish increasing incidence of disease and mortality and affecting productive performance. *Arapaima gigas* (pirarucu) is an Amazonian air-breathing and largest freshwater fish with scales in the world. The captivity development of pirarucu is expanding since it can fatten up over 1 kg per month reaching 10 kg body mass in the first year of fattening. This work was conducted in three periods (April to July 2010, August to November 2010, and December 2010 to March 2011) defined according to rainfall and medium temperatures. Seasonality effect analysis was performed on biochemical (lectin activity, lactate dehydrogenase, and alkaline phosphatase activities) and hematological (total count of red blood cells, hematocrit, hemoglobin, and hematimetric Wintrobe indexes) stress indicators, as well as on growth and wellbeing degree expressed by pirarucu condition factor developed in captivity. All biochemical and hematological stress indicators showed seasonal variations. However, the fish growth was allometrically positive; condition factor high values indicated good state of healthiness in cultivation. These results reinforce the robust feature of pirarucu and represent a starting point for understanding stress physiology and environmental changes during cultivation enabling identification and prevention of fish adverse health conditions.

## 1. Introduction

Pirarucu, *Arapaima gigas* (Shinz 1822), is the largest Amazonian and freshwater fish with scales in the world. It is considered an air-breathing fish and constitutes a species with great potential for farming due to interesting features such as excellent taste of meat and high growth rate with extraordinary weight development [1]; in captivity the fish can fatten up over 1 kg per month reaching 10 kg body mass in the first year of fattening [2]. Pirarucu has been a target of fishermen, due to its occurrence in lakes and isolated environments, which allows a disorderly intense exploration depreciating their natural stocks [3]. The expansion of the creation of pirarucu in captivity around the world, for commercial exploitation, was too an alternative to reduce the risk of extinction.

The increase of fish farming has developed a growing interest by producers in respect to the search for solutions to avoid the losses caused by mortality and production problems. Stress caused by common farming practices (acute stress) as well as environmental factors (chronic stress) such as seasonal changes increases the incidence of disease and mortality affecting the productive performance of animals [4]. The seasonal cycles can affect fishes in several biological activities, such as behavior, nutrition, metabolism, immunity, and reproduction [5]. The immunocompetence is often affected by seasonal variations and, in general, biochemical, hematological, and immunological parameters such as levels of various blood cells, hematocrit percentages [6], lysozyme activity [7], respiratory burst levels of head-kidney macrophages [8], and lectin activity [9]. The biochemical and hematological parameters are useful tools to determine the characteristics of fish blood in different situations such as stress or normality. The relative robustness or degree of wellbeing from a fish is expressed by the coefficient of condition or condition factor (CF), considered basically as the quotient between observed mass and theoretical mass estimated through the length-mass relationship. Variations in fish's coefficient of condition primarily reflect the state of sexual maturity and degree of nourishment; they provide relevant information about physiological and health characteristics of individual or population, which are very important in captive fish to their management and maintenance [10].

The aim of this work was to analyze the effects of seasonality (temperature and rainfall) on biochemical (lectin activity; lactate dehydrogenase; and alkaline phosphatase activities) and hematological parameters (hematocrit, hemoglobin, and hematimetric indexes of Wintrobe) that can be used as physiological indicators of stress. Also the influence of seasonality on growth of pirarucu developed in captivity as well as the state of wellbeing of the fish was analyzed. This is a first study that relates chronic stress with biochemical and hematological indicators of seasonal stress as well as health and growth in *A. gigas* fish farming.

## 2. Material and Methods

### 2.1. Weather Data Obtention: Rainfall and Temperature

**Figure 1:**
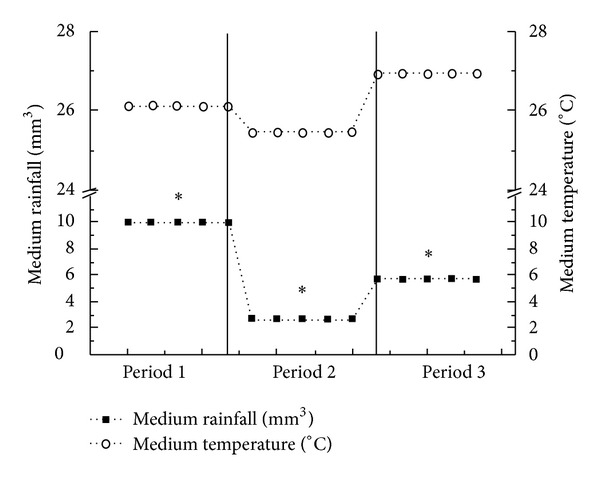
Medium rainfall and medium temperature for each period. Period 1 (April–July, 2010); Period 2 (August–November, 2010); Period 3 (December 2010 to March 2011). Data were obtained from *Instituto Nacional de Meteorologia* (INMET, Brazil). (*) Significant difference in rainfall among the periods (*P* < 0.05).

Weather data was provided by the *Instituto Nacional de Meteorologia* (INMET, Brazil), collected from weather station 82900 (08°03′S 34°57′W) Recife, PE, Brazil, according to international standards of the World Meteorological Organization. Medium rainfall (mm^3^) as well as medium temperature (°C) was calculated to each period, respectively: 9.99 mm^3^ and 26.14°C to period 1—P1 (April to July 2010); 2.74 mm^3^ and 25.46°C to period 2—P2 (August to November 2010); 5.8 mm^3^ and 26.94°C to period 3—P3 (December 2010 to March 2011) (Figure 1).

### 2.2. Obtaining Fish Blood

Fish were provided by the *Estação de Aquicultura Continental Prof. Johei Koike*, *Departamento de Pesca da Universidade Federal Rural de Pernambuco* (UFRPE) and developed in earth pond. The juvenile animals (*n* = 6) were anesthetized by hypothermia on ice; immediately after blood collection procedure the fishes returned to earth pond. Blood was obtained from caudal vein with syringes 5 mL, 21 G, 23 G, or 25 G needles (BD Precision Glide, PN, Brazil), according to fish size. To obtain whole blood tubes containing EDTA 1.8 mg/mL as anticoagulant (Vacuette, Greiner bio-one, Brazil) were used. Serum was collected from tubes without anticoagulant; the blood was centrifuged at 3000 ×g for 10 min at 4°C.

### 2.3. Lectin Activity and Protein Evaluation

Serum lectin activity (LA) was evaluated as specific hemagglutinating activity in microtiter plates with 96 wells [11]. Specific hemagglutinating activity was defined as the ratio between titer and protein concentration (mg/mL) and expressed with hemagglutinating activity units for protein milligrams (HAU/mg). Protein concentrations were determined by Bradford [12].

### 2.4. Lactate Dehydrogenase Activity

Lactate dehydrogenase activities (LDH) were determined following the oxidation of NADH (340 nm, 25°C). The reaction mixture contained a total volume of 1 mL, 50 mM imidazol, 1 mM KCN buffer pH 7.4 at 25°C, 0.13 mM of NADH, and different concentrations of pyruvate for LDH saturation plots. One unit of enzyme activity is defined as the amount of enzyme using 1 *μ*mol of substrate per min (340 nm, 25°C). Each value represents the mean of three measurements.

### 2.5. Alkaline Phosphatase Activity

The serum alkaline phosphatase activity (AP) was performed with modifications [13]. Briefly, enzyme activity was measured using p-nitrophenyl phosphate (pNPP) as substrate (5.0 mM) in 1 M diethanolamine (pH 9.8) containing 1 mM MgCl_2_ (405 nm, 25°C, 1 min).

### 2.6. Total Count of Red Blood Cells

To determine the total count of red blood cells (RBC), a 1 in 1000 dilution was made in 0.02 M phosphate saline buffer (PBS, pH 7.3). Counts were carried out using a Neubauer haemocytometer (INLAB, Brazil) and expressed as cell/mm^3^ [14].

### 2.7. Hematocrit, Hemoglobin, and Hematimetric Indexes of Wintrobe

The hemoglobin (Hb) levels were obtained using a kit for determination of hemoglobin in whole blood (Doles, Brazil) following the manufacturer's instructions. The hematocrit (Htc) was determined by the microhematocrit technique and result was expressed as percentage of erythrocytes compared to whole blood. Hematimetric indexes of Wintrobe were calculated as follows: MCV (mean corpuscular volume) = Htc/RBC × 10 (fl); MCH (mean corpuscular hemoglobin) = Hb/RBC × 10 (pg); and MCHC (mean corpuscular hemoglobin concentration) = Hb/Htc × 100 (g/dL).

### 2.8. Condition Factor

Mass (g) and length (cm) were used to determine CF and constant regression (*b*), which reveals the rate of growth in mass. Empirical point ratios *M*/*L* (mass/length) for each period were submitted to regression analysis and adjusted by power function, *M* = *aLb*, where *M* is the dependent variable, *L* is the independent variable, “*a*” is the CF, and “*b*” is the constant associated with the type of growth in mass of animals. These constants were estimated by linear regression of the transformed equation: *M* = log⁡*a* + *b* × log⁡*L*, where *M* = mass (g), *L* = total length (cm), *a* = constant, and *b* = growth exponent or constant regression [10].

### 2.9. Statistical Analysis

Data shown represent the mean values of each parameter in the specified periods. Statistical significance of data between groups (mean ± s.e.) was determined with analysis of variance (ANOVA) and Tukey test using OriginPro 8.0 (OrginLab Corporation, USA). A value of *P* < 0.05 was considered significant.

## 3. Results

**Figure 2:**
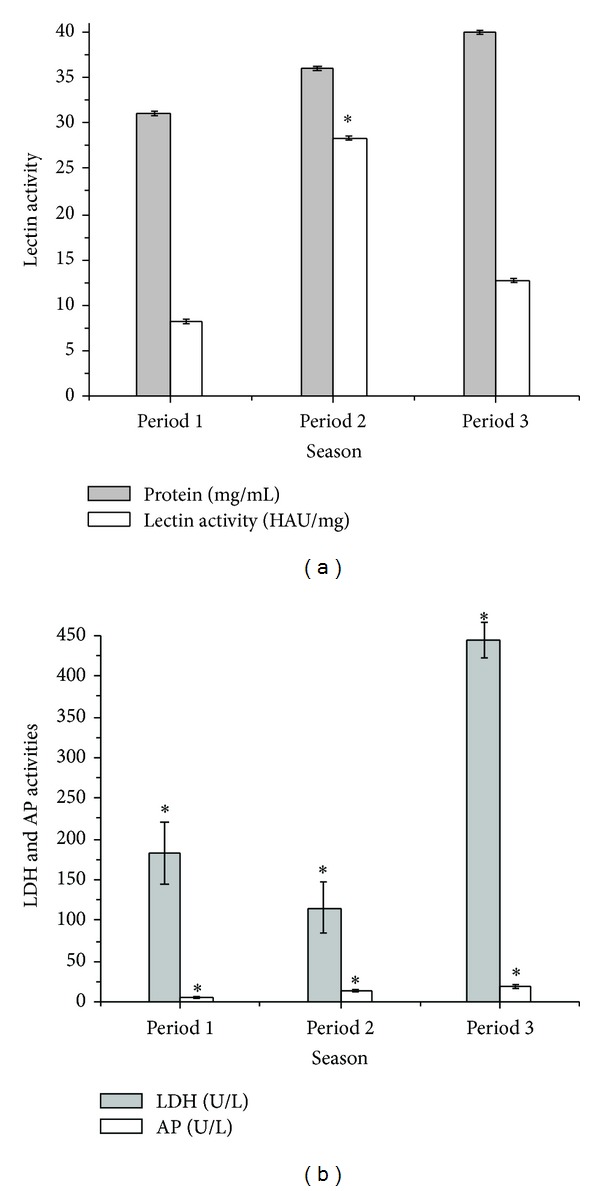
Variations in serum of *A. gigas* in Period 1 (9.99 mm^3^, 26.14°C), Period 2 (2.74 mm^3^, 25.46°C), and Period 3 (5.8 mm^3^, 26.94°C) to lectin activity (specific hemagglutinating activity) and serum protein concentration (a); LDH: lactate dehydrogenase activity and AP: alkaline phosphatase activity (b). (*) Significant (*P* < 0.05).

Significant difference was observed to serum lectin in the periods (*P* < 0.05); activity increased in P2 (28.45 HAU/mg) while in P1 (8.26 HAU/mg) and P3 (12.8 HAU/mg) values were lower, indicating some seasonal influence on LA (Figure 2(a)). The activity of the serum enzyme LDH was significantly higher in P3 (444.0 U/L) than in P1 (184.0 U/L) and P2 (115.0 U/L); in addition, there was a greater decrease in LDH activity in P2 than in P1 (Figure 2(b)). Serum AP activity in the studied periods showed a gradual increase (5.83 U/L P1, 13.0 U/L P2, and 18.0 U/L P3) and was significantly different from one period to another (Figure 2(b)).

**Figure 3:**
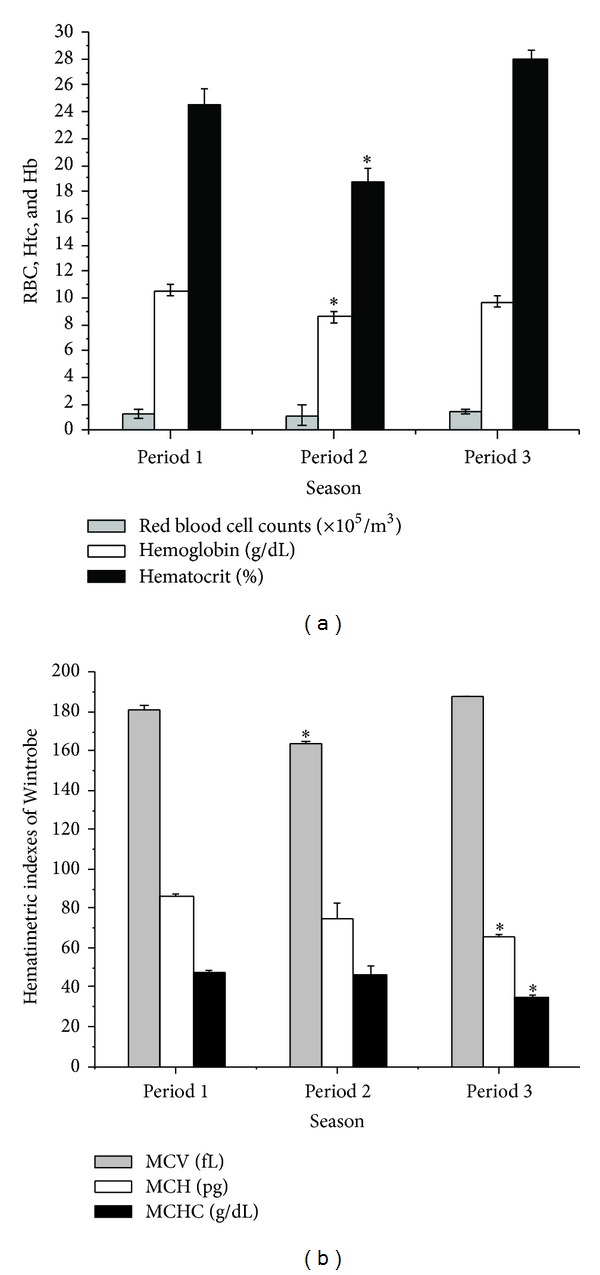
Variations in serum of *A. gigas* in Period 1 (9.99 mm^3^, 26.14°C), Period 2 (2.74 mm^3^, 25.46°C), and Period 3 (5.8 mm^3^, 26.94°C) to RBC: red blood cell counts, Hb: hemoglobin, and Htc: hematocrit (a); hematimetric indexes of Wintrobe—MVC, MCH, and MCHC (b). (*) Significant (*P* < 0.05).

RBC (1.24 × 10^6^/mm^3^ P1, 1.14 × 10^6^/mm^3^ P2, and 1.48 × 10^6^/mm^3^ P3), Hb levels (10.6 g/dL P1, 8.55 g/dL P2, and 9.7 g/dL P3), and Htc percentages (24.6% P1, 18.75% P2, and 27.9% P3) revealed values decreasing in P2; however this decrease was significant to Hb and Htc and not to RBC (Figure 3(a)). MCV showed seasonal variation with values significantly lower in P2 (198.4 fl P1, 164.4 fl P2, and 188.5 fl P3); no significant difference was showed between the other periods. MCH decreased gradually from P1 to P3 (85.5 pg P1, 75.05 pg P2, and 65.5 pg P3). MCHC (43.08 g/dL P1, 45.6 g/dL P2, and 34.75 g/dL P3) was significantly lower in P3, while P1 to P2 did not reveal significant difference (Figure 3(b)).

**Figure 4:**
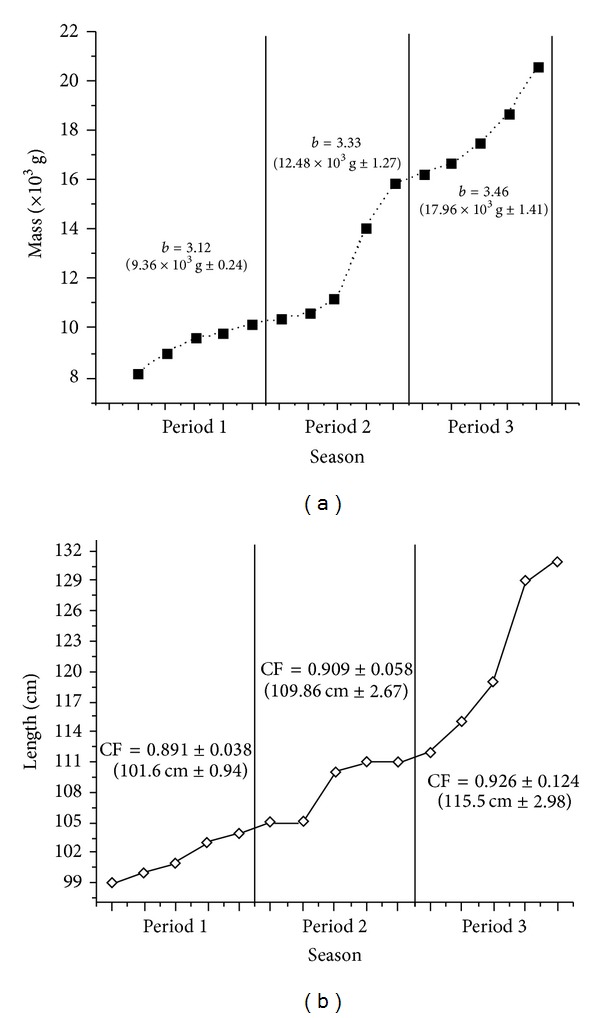
Variations in mass and estimated values for the regression constant “*b*”, rate of growth in mass (a); variations in length and condition factor (CF), (b) to *A. gigas* in Period 1 (9.99 mm^3^, 26.14°C), Period 2 (2.74 mm^3^, 25.46°C), and Period 3 (5.8 mm^3^, 26.94°C).

The constant values of linear regression “*b*” obtained in P1 (*b* = 3.12), P2 (*b* = 3.33), and P3 (*b* = 3.46) revealed a positive allometrically growth to pirarucu; this can be observed by a progressive increase in body mass in grams (9.36 × 10^3^ ± 0.24 P1, 12.48 × 10^3^ ± 1.27 P2, and 17.96 × 10^3^ ± 1.41 P3, mean values per period) throughout the periods (Figure 4(a)). The values of the constant linear regression “*b*” may vary from 2.50 to 3.50 [15]. This wide variation of “*b*” is a function of biotic and abiotic factors; when *b* = 3 growth is isometric, *b* > 3 is positive allometric, and *b* < 3 is negative allometric. Isometric growth (*b* = 3.00) suggests an increase in mass and length at the same rate which is theoretically ideal for fish, especially in cultivation. CF average showed high values, 0.891 ± 0.038 in P1, 0.909 ± 0.058 in P2, and 0.926 ± 0.124 in P3; progressive increase in fish length (cm) over the periods was observed (101.6 ± 0.94 P1, 109.8 ± 2.67 P2, and 115.5 ± 2.98 P3, mean values per period) (Figure 4(b)).

## 4. Discussion

Lectins are proteins or glycoproteins involved in innate immunity and are therefore considered as the first line of immune defense of fish [16]. These proteins have been found in serum, mucus skin, and eggs of several fish species [17, 18].

The seasonal variation found for *A. gigas* LA in P2 can be displayed in response to increased concentrations of specific potential pathogens in the environment due to lower rainfall and lowering temperature. Some pathogens have higher occurrence in winter, although the presence of pathogens generally is higher with increasing temperature, in summer [19]. Stress situations increased the levels of lectin; in this case it can function as an acute phase protein [20]. Seasonal variation too was found in activity of lectin present in serum of *Colossoma macropomum*, showing higher activity in summer and lower activity in winter [9].

The enzyme lactate dehydrogenase (LDH, EC: 1.1.1.27) has been approached in several studies with fish since it is directly linked to the glycolytic pathway and anaerobic metabolism, responsible for environmental stress responses. In this work the levels of LDH in pirarucu serum were significantly lower in P2 (*P* < 0.05) that demonstrates the relation with environmental change processes, such as changes in oxygen levels, even for air-breathing fish. The highest LDH levels were found in P3 showing that, probably, the enzyme activity in pirarucu is more sensitive to variation on temperature than on rainfall. Alkaline phosphatase (EC 3.1.3.1; AP) has been approached as a potential indicator of stress in the epidermal mucus of Atlantic salmon (*Salmo salar*); high levels of AP were observed in the mucosa of Atlantic salmon infected with the ectoparasite *Lepeophtheirus salmon* [21]. However, AP activity in pirarucu could be related with the fish growth. Human serum AP may provide an index of bone formation rate and probably plays a role in the mineralization of newly formed bone [22].

The seasonal variation observed in the RBC, Hb, and Htc in pirarucu may be due to a compensatory effect between rainfall and temperature. Thus, with more oxygen available in water, fewer red blood cells are needed to carry oxygen around the fish body; consequently, the hematocrit and amount of hemoglobin available are lower [23]. Similar results were obtained for RBC of *Oncorhynchus mykiss* [23], however with higher rates, probably due to higher seasonal variations in temperature experienced in the northern hemisphere, as well as the type of *A. gigas* breathing. Hematocrit values for *O. mykiss* were low in the summer (higher temperatures) and high in winter (lower temperatures) [24] demonstrating the differences in the physiological response to environmental stimuli into distinct species. Decrease in MCV in P2 is strongly related to low hematocrit percentage at this period indicating the possible development of anemia in response to seasonal variation, a chronic stress situation. The low Htc levels exhibit hemoconcentration as a help to cope with the stress related to oxygen demand, corroborating with the results obtained by Gomes [25]. Lower levels of dissolved oxygen should not be detrimental to the creation of pirarucu, especially juveniles, which during its development should provide breathing air, due to a change in its swim bladder [26]. This may be explained since *A. gigas* is considered an obligatory air-breathing fish; however, 10% of its breath still depends on the oxygen dissolved in water [24].

The type of growth observed for pirarucu was allometric positive (*b* > 3) indicating a greater increase in mass than in length. Growth-type isometric was observed to *A. gigas* in semi-intensive fish farming in central Amazonia [27]. Studies suggest that allometry should be used to characterize the different growth strategies of fish associated with ecological, behavioral and physiological characteristics [28]. CF is an index widely used in the bioecology of fish; it reflects the physiological status of the animal conditioned to interaction of biotic and abiotic factors [29]. The values of CF, in this work, did not show seasonal variation; however, the values were high indicating a good state of fish healthiness in cultivation. Furthermore, the progressive increase in CF having the highest value in P3 may be an indicator of onset gonadal development in *A. gigas* since it can also be used as an indicator of sexual maturation [10].

## 5. Conclusions

Lectin activity decreases in period of lower medium rainfall and temperature. LDH activity is more sensitive to changes in temperature than rainfall. AP could be related to bone growth of fish since length (cm), body mass (g), and enzyme activity increased progressively. Hb, Htc, and RBC decreased in response to dissolved oxygen in water and reflect changes in hematimetric indexes of Wintrobe. Pirarucu growth was allometrically positive; good healthiness in cultivation was indicated by CF.

Regulation of seasonal effects in fish has not yet been satisfactorily elucidated but surely constitutes a complex defense mechanism in these animals. *A. gigas* physiological information under stress caused by environmental factors, beside expanding the biological knowledge of the species, may also be useful in developing better techniques to increase the success of cultivation and improve fish production in different periods of the year.
